# The role of calcium channels in osteoporosis and their therapeutic potential

**DOI:** 10.3389/fendo.2024.1450328

**Published:** 2024-08-07

**Authors:** Ying Hao, Ningning Yang, Mengying Sun, Shangze Yang, Xingjuan Chen

**Affiliations:** ^1^ College of Sports, Northwest Normal University, Lanzhou, China; ^2^ Institute of Medical Research, Northwestern Polytechnical University, Xi’an, China

**Keywords:** osteoporosis, calcium ion channels, bone metabolism, calcium signaling, therapeutic targeting

## Abstract

Osteoporosis, a systemic skeletal disorder marked by diminished bone mass and compromised bone microarchitecture, is becoming increasingly prevalent due to an aging population. The underlying pathophysiology of osteoporosis is attributed to an imbalance between osteoclast-mediated bone resorption and osteoblast-mediated bone formation. Osteoclasts play a crucial role in the development of osteoporosis through various molecular pathways, including the RANK/RANKL/OPG signaling axis, cytokines, and integrins. Notably, the calcium signaling pathway is pivotal in regulating osteoclast activation and function, influencing bone resorption activity. Disruption in calcium signaling can lead to increased osteoclast-mediated bone resorption, contributing to the progression of osteoporosis. Emerging research indicates that calcium-permeable channels on the cellular membrane play a critical role in bone metabolism by modulating these intracellular calcium pathways. Here, we provide an overview of current literature on the regulation of plasma membrane calcium channels in relation to bone metabolism with particular emphasis on their dysregulation during the progression of osteoporosis. Targeting these calcium channels may represent a potential therapeutic strategy for treating osteoporosis.

## Introduction

Osteoporosis is a systemic skeletal disorder characterized by decreased bone mass and deterioration of bone microarchitecture, resulting in increased bone fragility and susceptibility to fractures (1, 2). According to a prevalence report encompassing 86 studies across five continents, the global prevalence rate of osteoporosis stands at 18.3%, with only 31-36% of individuals aged over 70 maintaining normal bone health. The remaining population exhibits varying degrees of osteopenia or osteoporosis (3). This number is expected to increase further due to the growing aging population. The incidence of osteoporotic fractures exceeds three to four times that of cardiovascular disease or cancer. A report in the US indicated that due to demographic changes, by 2040, approximately 25% of individuals over age 50 who have experienced hip fractures related to osteoporosis are expected to die within one year (4). The individual and societal impacts posed by osteoporosis and its complications present significant challenges for healthcare systems.

**Figure 1:**
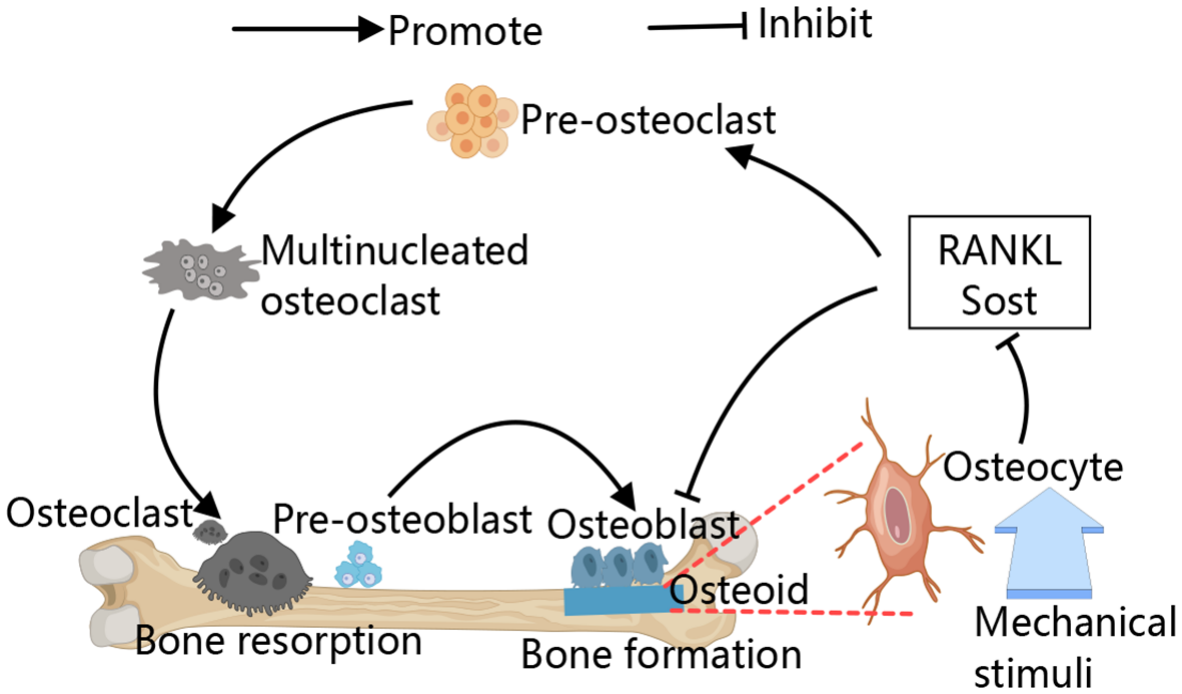
Schematic diagram of the bone remodeling. The process of bone remodeling begins with the recruitment of osteoclast precursors, which fuse to form multinucleated, active osteoclasts that mediate bone resorption. After resorption, osteoclasts leave the site, allowing osteoblasts to move in and cover the excavated area. Osteoblasts then initiate new bone formation by secreting osteoid. Osteocytes detect and respond to mechanical stimuli to regulate bone remodeling by regulating their secreted cytokines, for example, RANKL and sclerostin.

Primary osteoporosis includes postmenopausal and senile forms primarily attributed to declining estrogen levels and aging process (3, 5). Secondary osteoporosis is caused by underlying diseases or their treatments, including cardiovascular, neurological, endocrine disorders, and malignancies, as well as long-term glucocorticoid use, lifestyle factors, and major depression (6–9). Bone remodeling, a lifelong process, involves osteoclast-mediated bone resorption and osteoblast-driven bone formation (10–13). Additionally, osteocytes embedded within the bone matrix also play a role in this remodeling process, they are currently considered as the main source of molecules regulating the osteoclast and osteoblast activity, such as receptor activator of nuclear factor-κB ligand (RANKL) and sclerostin. Osteocytes detect and respond to mechanical and hormonal stimuli to coordinate both bone resorption and formation (14, 15). The primary pathological mechanism in osteoporosis is increased bone resorption due to abnormal osteoclast differentiation and proliferation (**Figure 1**) (16, 17). Consequently, treatment strategies focus on targeting osteoclast activity (18).

Additionally, recent studies suggest a correlation between bone microvessels and bone loss, with reduced trans-cortical vessels (TCV) observed in osteoporosis models and a positive correlation between TCV numbers and bone mass (19–21). Trans-cortical vessels are capillaries that run vertically through the cortical bone, connecting the endosteal and periosteal surfaces (20). Xiao CL et al. used old mice, ovariectomy mice, and db/db mice as osteoporosis models, finding reduced TCV in all models, which correlated positively with bone mass (20).

The regulation of osteoclasts formation and differentiation involves a complex interplay of cytokines, hormones, immune factors, gut microbiota, and cellular aging (22–25). Among the various cytokines and hormones that regulate osteoclasts formation and function, macrophage colony-stimulating factor (M-CSF) and RANKL are the most critical molecules. Senescent cells can produce senescence-associated secretory phenotypes (SASP), which exert deleterious paracrine and systemic effects, including the development of osteoporosis (26, 27). Comparative analysis between young mice (6 months old) and old mice (24 months old) revealed significantly higher expression levels of multiple SASP markers in osteocytes from the latter group (28). SASP has been reported to promote osteoclastogenesis by enhancing the survival of monocyte osteoclast progenitors. Moreover, inhibition of SASP using the JAK1/2 inhibitor Ruxolitinib has been shown to prevent age-related bone loss (29).

As a crucial intracellular second messenger, Ca^2+^ plays a significant role in the regulation of osteoclast differentiation and bone resorption (30). Many studies have shown that osteoclast dysfunction is often accompanied by increased intracellular calcium levels (17). Consequently, there has been a surge of interest in devising strategies to modulate the intracellular calcium system as a pivotal approach for regulating osteoclast function. Ca^2+^-permeable channels located on cell membranes are an essential component of the calcium signaling system, mediating the influx of extracellular Ca^2+^. Recently, accumulating evidence has highlighted the critical role these channels play in maintaining dynamic bone metabolism. In this review, we present a summary of existing research on the control of calcium channels in the plasma membrane concerning bone health, with specific focus on their irregularities during the development of osteoporosis. The exploration of these calcium channels as a potential therapeutic approach for managing osteoporosis is discussed.

## Calcium signaling in the process of bone remodeling

Calcium signaling plays a crucial role in bone metabolism, with imbalanced Ca^2+^ homeostasis significantly affecting osteoblast activity and bone formation. Excessive Ca^2+^ loading in osteoblasts can limit differentiation by inducing apoptosis in mitochondria and the endoplasmic reticulum (ER) (31, 32). Mitochondria are essential for normal bone formation; however, elevated intracellular calcium levels can disrupt the function of the inner mitochondrial membrane, thereby impairing bone formation (33, 34). Similarly, high intracellular calcium levels can cause ER stress, leading to apoptosis in osteoblasts (35). Bone marrow mesenchymal stem cells (BMMSCs) differentiate into osteoblasts and regulate osteoclast activity through the secretion of RANKL and osteoprotegerin (OPG), maintaining bone metabolism balance. BMMSCs play a crucial role in bone remodeling by directly forming new bone and indirectly influencing bone resorption. Li et al. reported disrupted intracellular calcium homeostasis in bone samples from osteoporosis patients and mice, leading to impaired osteoblast differentiation and compromised bone formation (36).

**Figure 2:**
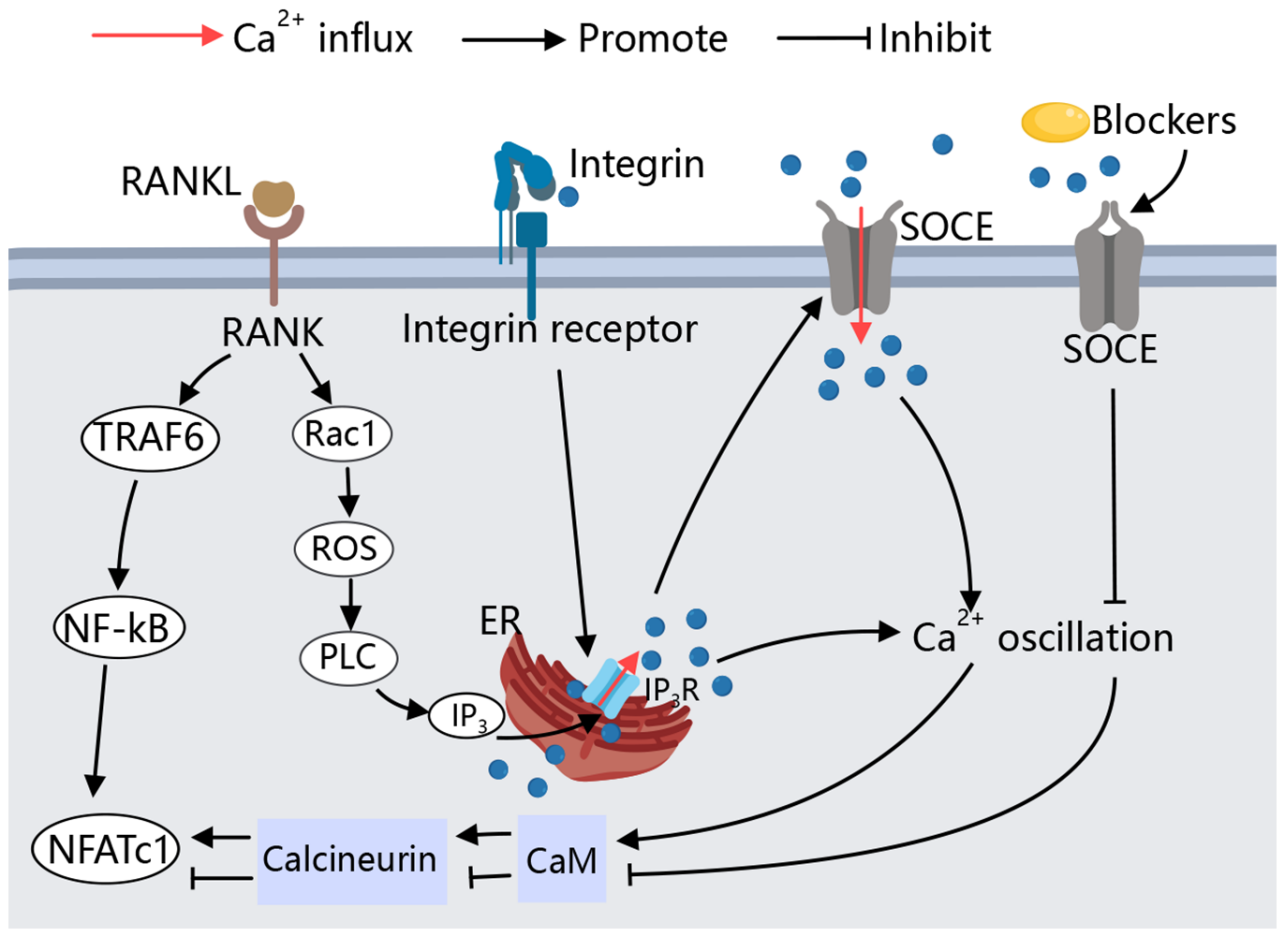
Calcium signaling in osteoclast. During RANKL-mediated osteoclastogenesis, cytoplasmic Ca^2+^ oscillation, Causing downstream related pathways to activate NFATC1,and thus inducing osteoclast differentiation. SOCE is involved in RANKL-induced Ca^2+^ oscillations and maintains the sustained production of Ca^2+^ oscillations, the Ca^2+^ oscillations induced by RANKL are blocked by SOCE blockers. Specific peptide activation of integrin receptors also causes a transient Ca^2+^ response, allowing osteoclasts to adhere to the bone matrix.

In osteoclast differentiation and function, calcium signaling is key (37) (**Figure 2**). RANKL utilizes this pathway to activate NFATC, promoting osteoclast formation (38). RANKL/RANK signaling transactivates phospholipase C (PLC), producing inositol 1,4,5-trisphosphate (IP_3_), which binds to inositol 1,4,5-trisphosphate receptors (IP_3_Rs) on the ER, triggering Ca^2+^ oscillations. This leads to Ca^2+^ binding to calmodulin, activating calcineurin, which dephosphorylates NFATc1, thus promoting osteoclast differentiation (39–41). Additionally, Kim et al. have identified an upstream signaling pathway for RANKL-induced PLC activation and Ca^2+^ oscillations. The small G protein Rac1 is the most upstream component activated by RANKL, and its activation can induce long-term production of reactive oxygen species (ROS) and trigger calcium oscillation by activating PLC to promote osteoclast differentiation (42, 43). However, continuous supply of Ca^2+^ is required for Ca^2+^ oscillations to reload the stores between spikes. RANKL-induced ER Ca^2+^ release occurs through the activation of STIM1 on the ER membrane, leading to STIM1 aggregation into puncta at the ER-PM junction. This induces an influx of extracellular calcium via store-operated calcium entry (SOCE), facilitating osteoclast genesis (42–44). Both types of Ca^2+^ oscillations are terminated when extracellular Ca^2+^ removing or SOCE blocker Gd3+ are used (43). RANK-bound RANKL also activates TRAF6, leading to NF-κB activation and NFATc1 transcription (45, 46). Studies on (–)-Epicatechin 3-O-β-D-allopyranoside (ECAP) show it inhibits osteoclastogenesis by blocking NF-κB activation and reducing NFATc1 expression (47).

In the absence of cellular calcium Ca^2+^, integrins are also able to activate intracellular Ca^2+^ storage structures by binding to integrin receptors at the cell membrane, induced an increase in intracellular Ca^2+^ (48). IP_3_Rs, particularly IP_3_R2, regulate calcium release from the ER, playing a critical role in osteoclast genesis (49). In cultured osteoclasts and monocyte precursors, increased intracellular Ca^2+^ lead to decreased adhesion and reduced expression of podosomes, key to osteoclasts function (50).

Maintaining proper intracellular Ca^2+^ levels is essential for osteoclast differentiation and activity, making calcium signaling pathways potential therapeutic targets for osteoporosis. The calcium permeability channels in the cell membrane are critical for extracellular calcium influx, which is vital for bone metabolism. An imbalance in calcium homeostasis due to these channels can impact the function and differentiation of both osteoblasts and osteoclasts.

## Calcium channels in osteoclasts

Calcium channels influence skeletal homeostasis by mediating processes that mediate the extracellular and intracellular Ca^2+^ balance, Osteoclasts differentiation and apoptosis are widely regulated by Ca^2+^ channels located in the cell membrane. The regulation of these Ca^2+^ channels on osteoclasts is detailed below (**Table 1**).

**Table 1:** Calcium channels and their functions in osteoblasts and osteoclasts.

Channel type		Osteoblasts/ osteoclasts	Mechanism	Physiological/Pathological outcome	Ref.
VGCC	L&T	Promoting OB differentiation		Upregulation: improve osteoporosis Downregulation: decreased OB differentiation	(51, 52)
	VGCC	Promoting OC differentiation	Stimulation elevated the channel activity	Downregulation: decreased Ca^2+^ activity	(50)
SOCE	STIM1	Promoting osteoclast differentiation	Keep calcium oscillation continued	Downregulation: Calcium oscillation	(43, 53, 54)
	ORAI1	Promoting osteoclast differentiation	Combined with STIM 1 to maintain calcium oscillation	Downregulation: decreased Ca^2+^ influx, NFATC1 translocation injury, and decreased osteoclast formation	
SOCE	STIM1	Promoting OC differentiation	Keep calcium oscillation continued	Downregulation: Calcium oscillation	(43, 53, 54)
	ORAI1	Promoting OC differentiation	Combined with STIM 1 to maintain calcium oscillation	Downregulation: decreased Ca^2+^ influx, NFATC1 translocation injury, and decreased OC formation	
TRPs	TRPC1	Promoting OC differentiation		Upregulation: increased OC differentiation and decreased bone mass	(55)
	TRPC3	Promoting OC differentiation		They are complementary relationships	(56)
	TRPC6				
	TRPV1	Promoting OB and OC differentiation		Loss of TRPV1:osteoclast differentiation↓, increased bone mass; BMMSCs differentiation↓ osteogenesis↓	(57, 58)
	TRPV5	Promoting OC differentiation		Knockout number and volume↑, bone resorption↓. However, the bone resorption was enhanced in the mature	(59–61)
	TRPV6	Inhibition OC differentiation	Inhibition of the IGF IR-PI3K-AKT pathway	Knockout number and volume↑, bone resorption↑.	(17, 30)
	TRPM7	Promoting OB differentiation		Downregulation differentiation and mineralization↓	(62)
	TRPM8	Promoting OB differentiation	promotes the differentiation of BMMSCs into OB	Downregulation: OB differentiation ↓	(63)
	TRPML1	Promoting OC differentiation			(64)
P2X	P2X1	Promoting OB differentiation			(65)
	P2X4	Promote OB and OC differentiation		Downregulation: OB differentiation↓ OC differentiation↓	(66)
	P2X7	Activation of OC		Osteoporosis can activate P2X7 and OC differentiation increases	(67)
IP_3_Rs	IP_3_R2	Promoting OC differentiation		Loss of IP_3_R2: diminished OC differentiation	(49)
Piezo	Piezo1	Promoting OB differentiation		knockout: OB differentiation ↓	(68, 69)

IGFIR-PI3K-AKT, Insulin-like growth factor insulin receptor-phosphatidyl inositol 3-kinase-protein kinase; BMMSCs, Bone marrow mesenchymal stem cells; OB, Osteoblast; OC, Osteoclast; ↑: indicates that the functions have been increased; ↓: indicates that the functions have been weakened.

### Voltage-gated calcium channels

Most of the studies on VGCCs have focused on excitable cells such as neuro- and muscle cells. However, an increasing body of research has also demonstrated the involvement of VSCCs in nonexcitable cells, including bone cells and stem cells (70–72). These studies have highlighted the crucial role of VSCCs in bone remodeling. Specifically, disrupting VSCCs or using blockers can significantly impair osteogenesis and result in abnormal bone metabolism (73). The depolarization of the membrane potential activates VGCCs, mediating the influx of extracellular calcium (74). The Ca^2+^ influx of VGCCs was monitored in cultured osteoclasts of chickens by their depolarization to the membrane. Found that the enhanced activity of VGCCs in stimulated osteoclasts could affect cell adhesion and reduce bone resorption activity (50).

### Store-operated calcium entry

SOCE is considered as the primary pathway for calcium release-activated calcium (CRAC) channel activation, involving the ORAI1 channel on the plasma membrane (PM) and the stromal interaction molecule 1 (STIM1) on the ER membrane. When ER calcium ion levels are high, STIM1 remains inactive. However, reduced ER Ca^2+^ levels activate both STIM1 and STIM2, triggering conformational changes in STIM1, its translocation to the PM, redistribution to ER-PM junctions, interaction with clustered Orai1 channels, and subsequent extracellular calcium influx into cells (75, 76). This process is crucial for maintaining intracellular calcium homeostasis during cellular activation by external stimuli. STIM1 knockout in precursor osteoclasts attenuates Ca^2+^ oscillations induced by RANKL (43), and ORAI1 knockdown impairs bone mineral resorption and leads to osteoclast deficiency, indicating that SOCE is essential for the Ca^2+^ oscillation/NFATc1-dependent signaling complex induced by RANKL (53, 54).

### Transient receptor potential channels

TRP channels, comprising six subfamilies (TRPC, TRPV, TRPP, TRPM, TRPA, and TRPML), play significant roles in maintaining Ca^2+^ balance during bone homeostasis (77, 78). TRPC1 inactivation under physiological conditions in mice, and the subsequent upregulation of osteoclasts in MyoD family isoform “A” (*I-mfa*) knockout mice, suggests the role of TRPC1 in osteoclast differentiation (55). TRPC3 and TRPC6 are associated with osteoporosis, with TRPC3 promoting osteoclast differentiation and bone resorption in TRPC6-deficient phenotypes (56). TRPV channels are extensively documented in osteoblast and osteoclast differentiation. TRPV1 promotes osteoclast differentiation, with TRPV1-deficient mice showing reduced osteoclast numbers (57, 58). TRPV2 facilitates Ca^2+^ oscillation during osteoclast genesis (79), and TRPV4 is crucial during late osteoclast differentiation stages (80, 81). TRPV5 and TRPV6 regulate osteoclast size and number, with TRPV6 acting as a negative regulator (30, 59–61). Knockdown of TRPV6 resulting in a significant rise in bone resorption (17). Both knockdown and deletion of TRPML1 significantly reduced the differentiation of bone marrow-derived macrophages into osteoclasts (64), highlighting the complex roles of TRP channels in osteoclast function.

### Others

Ryanodine receptors (RyRs) and IP_3_Rs mediate the release of Ca^2+^ from the endoplasmic reticulum (ER). The activation of PLC leads to the production of IP_3_, which subsequently binds to IP_3_Rs on the ER membrane, causing the release of Ca^2+^ stores. Gene knockout studies suggest that IP_3_R2 plays a critical role in calcium oscillation during osteoclastogenesis (49), with its absence resulting in a partial defect in osteoclast differentiation (82). RyRs calcium channels may contribute to the release of intracellular calcium stores, while plasma membrane-bound RyR2 potentially regulates osteoclast activity based on extracellular calcium concentration (83).

Purinoceptor 2X (P2X) receptors are ligand-gated ion channels primarily activated by ATP and exhibit significant permeability to Ca^2+^ (84). The P2X4 receptors are highly expressed in both osteoblasts and osteoclasts, and their inhibitors have been shown to dose-dependently inhibit osteoclastic and osteogenic differentiation (66). Studies examining OPG induced osteoclast adhesion structural changes mediated by mitogen-activated protein kinase (MAPK) signaling via P2X7 receptors indicate that loss of P2X7 receptors inhibits osteoclast activation (67).

Despite the large number of studies demonstrating the regulation of calcium channels in osteoclasts, Compared with mesenchymal osteoblasts and osteocytes, few studies regulate osteoclasts by VGCC, with unclear regulatory mechanisms likely due to the lower sensitivity of VGCCs in non-excitatory cells. Both ORAI1 and STIM1 have shown positive regulation of osteoclasts in the SOCE pathway. The regulation of osteoclast differentiation and activity by the TRP family, particularly TRPC and TRPV channels, is well-documented. Existing studies support the negative regulatory role of the TRPC family on osteoclasts. Additionally, TRPC3 has been found to be highly expressed in individuals with reduced bone mass (56), suggesting that TRPC3 may serve as an early warning signal for osteoporosis. The TRPV family is notably significant in regulating osteoclasts, with its six members influencing osteoclast differentiation and function. This family presents a potential target for osteoporosis treatment, though specific targets within the TRPV channels have yet to be identified. Future research should focus on discovering precise targets within TRPV channels and identifying new Ca^2+^ channels that could serve as therapeutic targets for osteoporosis.

## Calcium channels in osteoblasts

**Figure 3:**
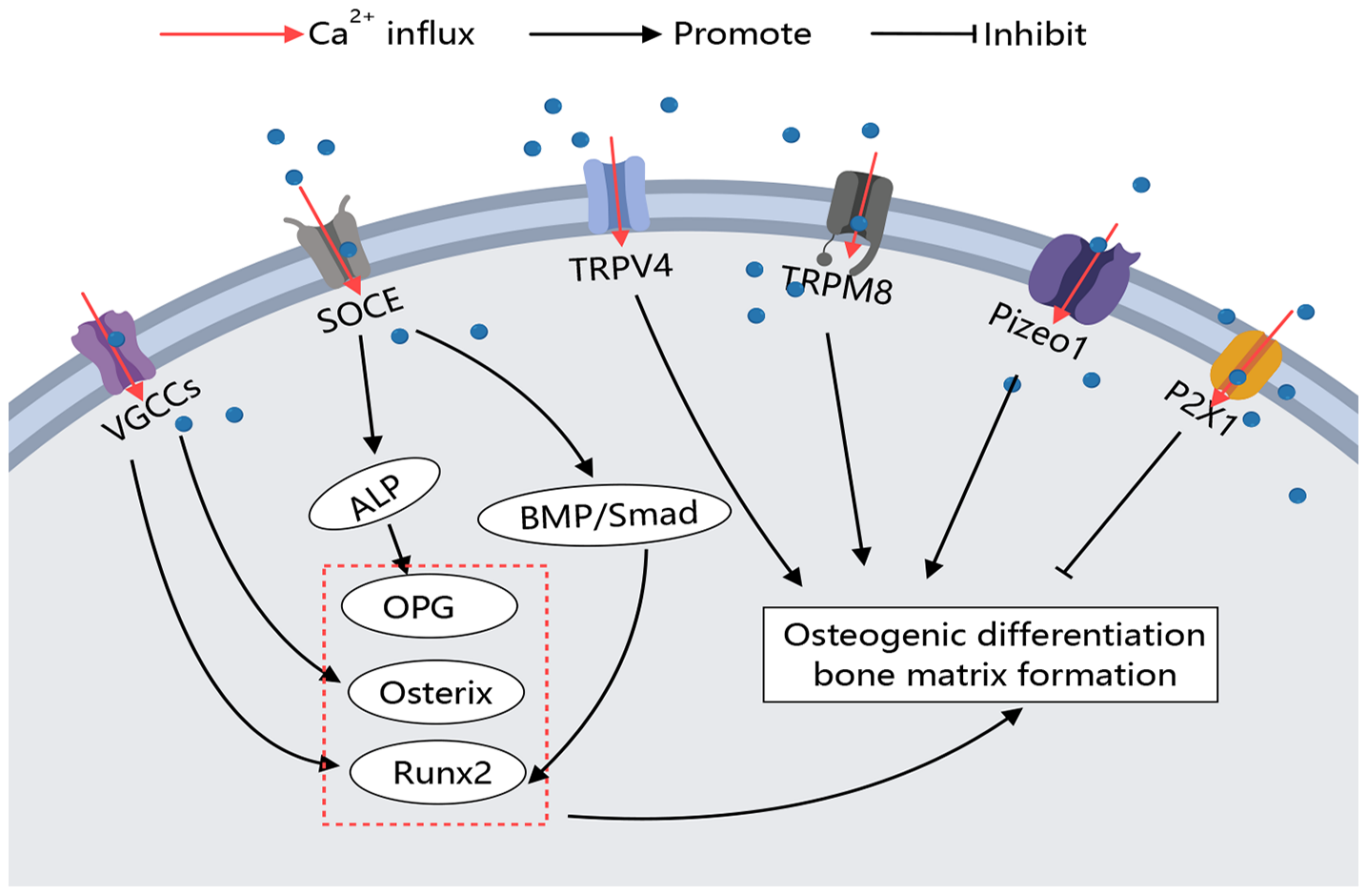
Calcium Channels in Osteoblasts. Various calcium channels on the osteoblast membrane play crucial roles in regulating osteoblast activity. Different types of calcium channels can modulate bone-forming factors, thereby promoting or inhibiting osteoblast differentiation and function through distinct signaling pathways.

The activation of calcium channels on the osteoblast membrane plays a crucial role in regulating cell differentiation and activity, with varying regulatory processes and effects observed across different types of calcium channels. The regulation of these Ca^2+^ channels on osteoclasts is outlined as follows (**Figure 3**).

### VGCCs

The calcium increase caused by bone microinjury area induced the formation of bone matrix. The main mechanism is that extracellular high calcium stimulated cell membrane depolarization, which enabled Ca^2+^ to enter MC3T3-E1 cells in local areas through L-type and T-type VGCC and promoted bone matrix formation. When L and t-type blockers were used, the calcium inflow activity of cells in the bone micro-damage area was significantly reduced (51). Osteoblasts of the mouse skull were seeded on inactivated bovine bone wafers, and by inducing local diffuse damage near the cells, the cells on the damaged bone wafers showed significant increases in Runx2 and Osterix expression and synthesis of numerous osteocalcin and mineralized nodules. Moreover, when cells were treated with the nonselective VGCC inhibitor bepredil before loading, Runx2 and Osterix expression were significantly inhibited and osteocalcin and mineralized nodule formation was significantly reduced, indicating that the diffuse microlesion-induced Ca^2+^ efflux activates the anabolic response in osteoblasts by activating VGCCs (52). Functional mutated Ca_V_1.2 mice exhibited elevated serum concentrations of OPG, while isolated BMMSCs displayed a reduced ratio of RANKL to OPG (85). Fei D et al. and Zhang Y et al. identified the downregulation of CaV1.2 in Zmpste24-mouse BMMSCs as a limiting factor for osteogenic differentiation.

In contrast, pharmacological upregulation of Ca_V_1.2 activity alleviated osteoporosis in *Zmpste24^-/-^* mice (86). Bei Li et al. discovered that alkaline phosphatase (ALPL) regulates L-type calcium channel trafficking by binding to the α2δ subunit to maintain intracellular calcium homeostasis. Reduced intracellular calcium levels due to alkaline phosphatase deficiency lead to decreased osteogenic differentiation of BMMSCs, but ionomycin can improve the osteoporotic phenotype in *alpl^-/-^* mice and BMMSC-specific conditional *alpl^-/-^* mice by promoting L-type channel calcium flux (87).

### SOCE

In the context of SOCE, ORAI1 deficiency leads to significant reductions in Ca^2+^ influx, alkaline phosphatase activity, substrate mineralization, and overall bone formation (51). Studies have indicated that long-term usage of lansoprazole (LPZ) induces calcium overload in osteoblasts and triggers apoptosis. This calcium release primarily occurs through the store-operated calcium entry (SOCE) influx at the cell membrane and via the IP_3_Rs located on the endoplasmic reticulum. Inhibition of IP_3_Rs and SOCE pathway using 2-APB improves osteoporosis condition (32). Furthermore, ORAI1 has been identified as a crucial mediator for enhancing the osteogenic potential of BMMSCs. The absence of ORAI1 results in limited phosphorylation of Smad 1/5/8 within the BMP signaling pathway; however, activation of BMP signaling can rescue the impaired osteogenic differentiation ability observed in ORAI1 BMMSCs. These findings suggest that targeting ORAI1-BMP signaling could be a potential therapeutic approach for treating bone formation defects (88).

### TRPs

*In vitro* studies on TRPV1-deficient BMMSCs have demonstrated impaired osteoblast differentiation and mineralization (57). *In vitro* studies on TRPV1-deficient BMMSCs have demonstrated impaired osteoblast differentiation and mineralization (89). Although research on TRPM channels is limited, it has been shown that TRPM7 is upregulated during osteoblast differentiation, and its deficiency impairs osteoblast proliferation, differentiation, and mineralization (62). Notably, TRPM8 knockout mice exposed to cold temperatures exhibit reduced bone density and significant reductions in femoral size in males, along with lower vertebral bone microarchitectural parameters in females, suggesting TRPM8’s role in bone modeling and remodeling (90). Furthermore, TRPM8 promotes the differentiation of BMMSCs into osteoblasts. Treatments using TRPM8 agonists such as menthol or icilin enhance osteogenic differentiation, while the antagonist BCTC decreases it (63).

### Others

P2X1 receptors negatively regulate osteoblast mineralization (65). Recently discovered mechanosensory ion channels Piezo1 and Piezo2 also play a regulatory role in osteoblasts (68). Targeted deletion of Piezo1 in osteoblasts resulted in severe osteoporosis and spontaneous fractures, highlighting Piezo1’s function in growth plate chondrocytes (69). Additionally, the activation of Sr^2+^ on the calcium-sensing receptor (CaR) exerts an anti-osteoporotic effect. Compared to Sr^2+^, CaR has a better sensitivity to Ca^2+^, and the combination of Ca^2+^ and CaR can better promote osteoclast apoptosis and osteoblast differentiation, thereby enhancing bone tissue (91).

The regulatory effect of calcium channels on osteoblasts primarily focuses on the positive regulation of osteogenesis by VGCCs. L-type and T-type VGCCs are widely present on osteoblasts and are activated by changes in the extracellular environment, such as high calcium levels and hormones, promoting osteogenic differentiation. Additionally, TRPM8 has been shown to promote BMMSC osteogenic differentiation, although the specific mechanisms require further study. Through these findings, it can be observed that the activation of VGCCs, TRPs, SOCE, and Piezo channels on the osteoblast membrane can reduce the symptoms of osteoporosis (**Figure 3**).

## Calcium channels in osteocytes

**Figure 4:**
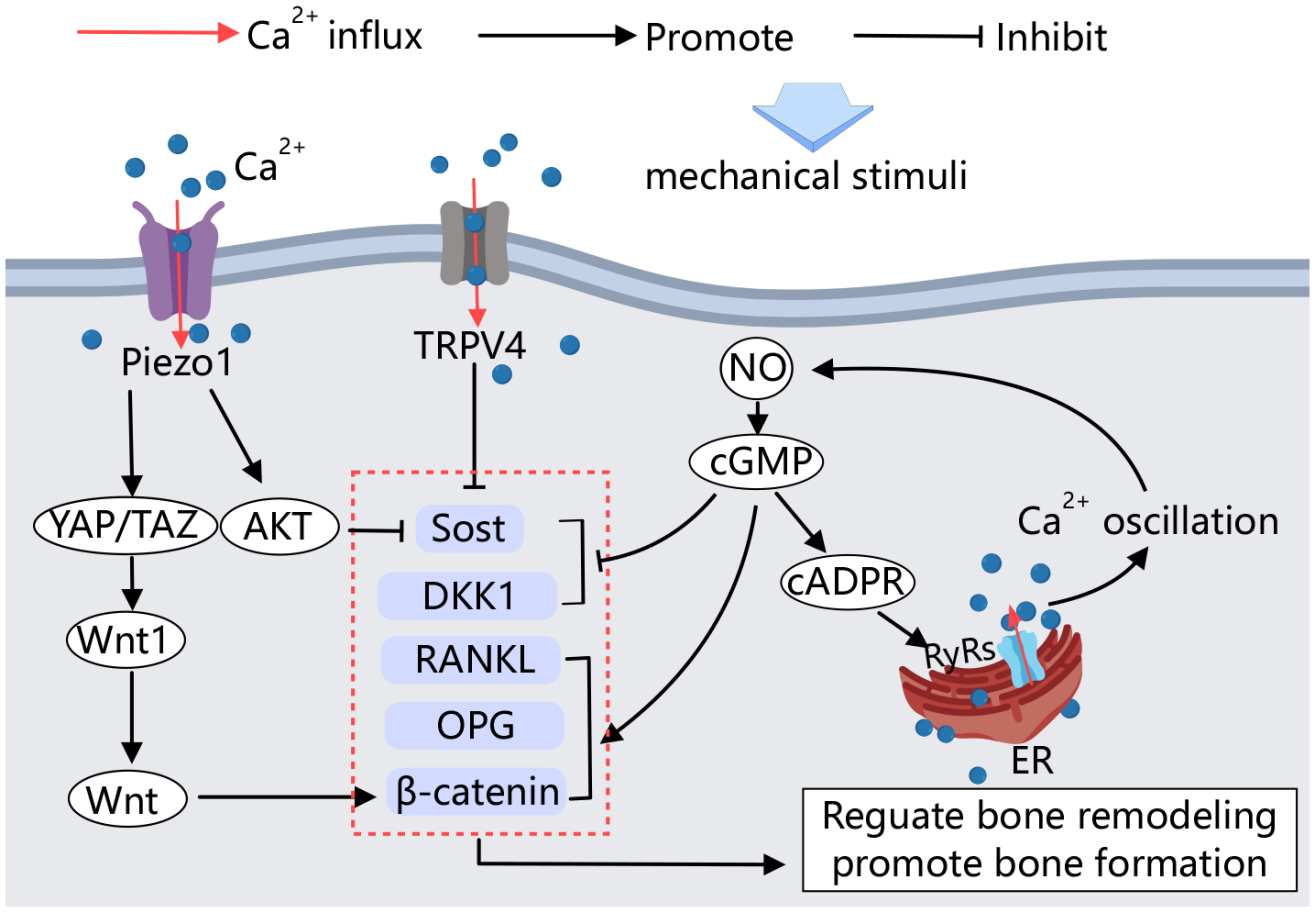
Calcium channels in osteocytes. Mechanical stimulation acting on osteocytes activates Piezo1 and TRPV4, influencing the activity of factors involved in bone formation and resorption, thereby regulating bone remodeling. Additionally, calcium signaling plays a crucial role in this process. Upon mechanical stimulation, an amplified positive feedback loop of Ca^2+^ signaling is established, further regulating bone remodeling.

Osteocytes are mechanical load-sensing cells that mediate bone formation, adaptation, and resorption in response to mechanical load (92). Various mechanical stimuli, including fluid shear stresses and matrix strains (e.g., compressive, tensile, and torsional loads), can activate osteocytes within the pericellular matrix (93). In the bone microenvironment, osteocytes are encased in a pericellular matrix at the interface between the cell membrane and the hard bone matrix. Small force stimuli generate fluid shear from the extracellular fluid flow due to spatial deformation, which activates mechanically stimulus-sensitive ion channels and integrins on the osteocyte membranes. This activation initiates a series of downstream pathways to regulate bone remodeling (**Figure 4**).

Several studies have demonstrated that mechanosensitive calcium channels in the osteoblast membrane are involved in the sensing of mechanical signals by osteocytes. Calcium influx is an early response to mechanical stimulation of osteocytes both *in vitro* and *in vivo* (94). The Ca^2+^ channel Piezo1 mediates mechanical signaling in osteocytes. Fluid shear stress has been shown to increase the expression of Piezo1 on the MLO-Y4 cell membrane and elevate intracellular calcium concentration. Conditional knockdown of Piezo1 in osteocytes leads to significant reductions in bone mass and impaired bone structure and strength in mice (95). Educed Piezo1 expression weakens the MLO-Y4 response to external stress. Activation of the Piezo1 calcium channel by the Piezo1-specific agonist Yoda1 produces effects similar to those observed in mechanically stimulated osteocytes (96).

Moreover, YAP1/TAZ has been identified as key mediators of sensory transmission to mechanical signals in various cell types. Mechanical stimulation of Piezo1 on osteocyte membranes activates YAP1 and TAZ, increasing Wnt1 production by osteoblasts (96). Wnt1 production, in turn, activates the Wnt/β-catenin signaling pathway, promoting bone formation (97). *In vitro* cell culture studies have shown that Piezo1 can also regulate the biological behavior of osteocytes through the Akt/Sost pathway. Sost is a key of bone regulator produced by osteocytes, inhibits the classical Wnt signaling pathway, thereby regulating bone formation. This signaling pathway stimulates resorption (98). High expression of Piezo1 in osteocytes, stimulated by mechanical stretching, immediately induces calcium efflux and Akt phosphorylation, which inhibits Sost expression, promotes bone formation, and inhibits bone resorption (99).

TRPV4 calcium channels have also been shown to inhibit Sost expression *in vitro* by mechanically stimulating osteocytes (100). Mechanical stimulation can regulate the transcription of bone cytokines through the NO-Ca^2+^ positive feedback signaling pathway in osteocytes (101). NO is an important signaling molecule secreted by osteocytes under mechanical loading. It regulates the downstream cGMP signaling pathway, which influences the transcription of cellular factors such as RANKL, OPG, and DKK1, β-catenin, and causes intracellular Ca^2+^ oscillation through the NO-cGMP-cADPR-RyRs pathway. This Ca^2+^ oscillation further promotes NO formation through a positive feedback loop, thereby regulating bone remodeling and promoting osteoblast angiogenesis (101–103).

## Conclusions and prospects

Osteoporosis, a serious age-related disease, poses a global challenge. Bone metabolism in osteoporosis is closely linked to the expression of Ca^2+^ channels in osteocytes, making their regulation crucial for managing the disease. TRP channels, known for their high calcium permeability, have several members significantly associated with osteoporosis. While research on TRPM8 has largely focused on tumors, cardiovascular diseases and pain (104, 105), its role in bone metabolism remains underexplored, particularly its impact on osteoclasts. Developing new therapeutic targets to address bone loss is essential, as current osteoporosis treatments often have side effects such as constipation, diarrhea, tumorigenesis, and cardiovascular disease. Further understanding of calcium channels in osteocytes, especially osteoclasts, could lead to healthier regulatory measures and new treatment ideas for osteoporosis.

Postmenopausal osteoporosis is the most common form of the disease, driven by significantly reduced estrogen levels, which lead to decreased bone mass, damaged bone microstructure, and higher fracture risk. Estrogen decline promotes osteoclast activity through mechanisms like upregulating RANKL and regulating microRNA-21 biogenesis, while also inhibiting osteogenic differentiation via increased TNF-α activity (106, 107). The specific interaction between estrogen and calcium ion channels remains unclear, presenting a promising direction for future research.

## Author contributions

YH: Writing – review & editing, Writing – original draft, Visualization, Validation, Supervision, Resources, Project administration, Methodology, Funding acquisition, Data curation, Conceptualization. NY: Writing – review & editing, Writing – original draft, Visualization, Software, Resources, Project administration, Methodology, Investigation, Formal analysis, Data curation, Conceptualization. MS: Writing – original draft, Visualization, Supervision, Software, Methodology, Investigation, Formal analysis, Data curation. SY: Writing – review & editing, Visualization, Supervision, Software, Data curation. XC: Writing – review & editing, Writing – original draft, Validation, Supervision, Resources, Project administration, Methodology, Funding acquisition, Conceptualization.
